# Supplementary figures and images for: Analysis of lung transcriptome in calves infected with Bovine Respiratory Syncytial Virus and treated with antiviral and/or cyclooxygenase inhibitor
Source: PLoS One. 2021 Feb 18;16(2):e0246695. doi: 10.1371/journal.pone.0246695 (PMC7891793; doi:10.1371/journal.pone.0246695)

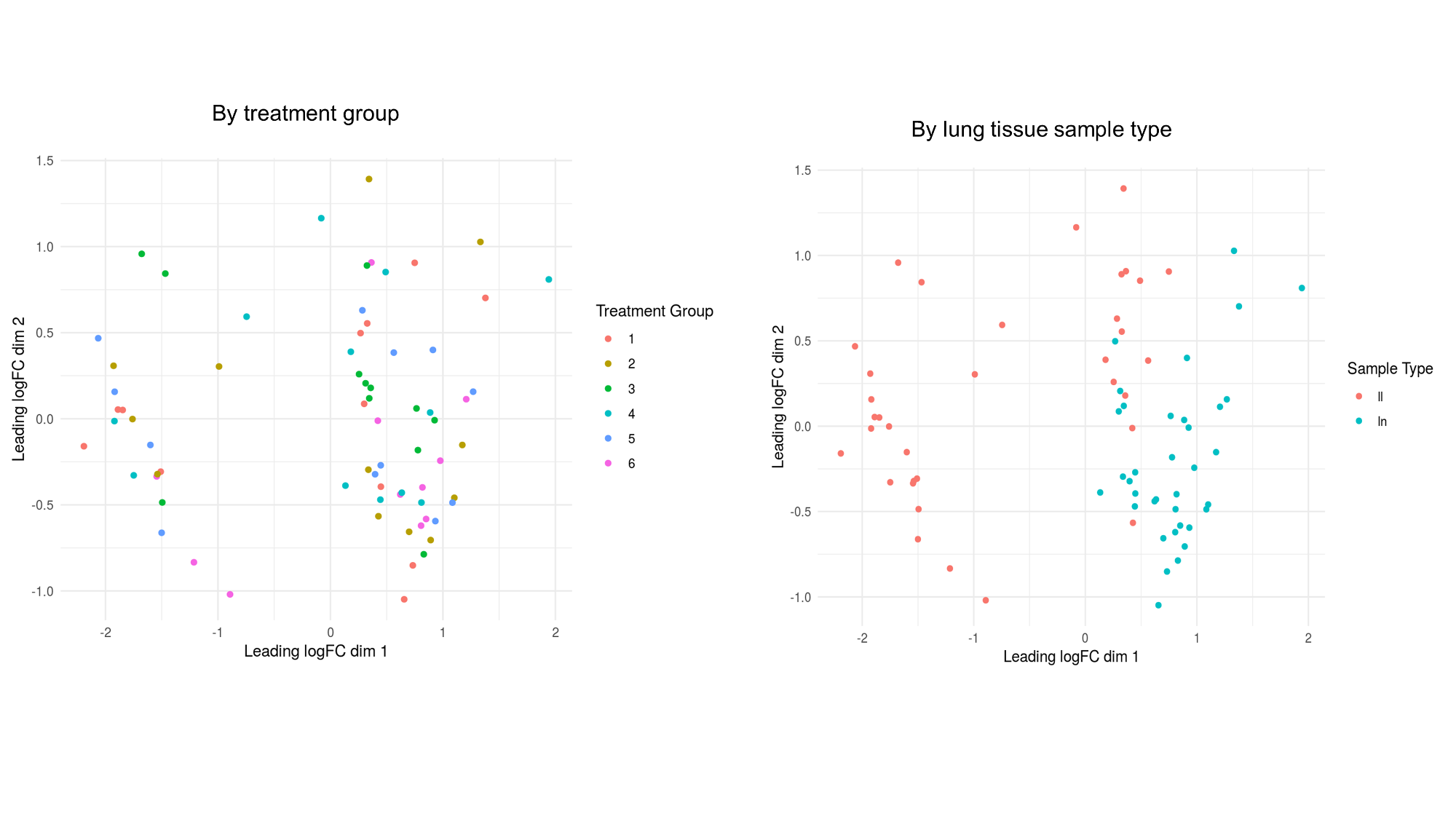

Supplement: S1 Fig — When data sets were compared by lung tissue sample type; LL appeared on the area that was distinct from the area of LN. (TIF) [file pone.0246695.s001.TIF]

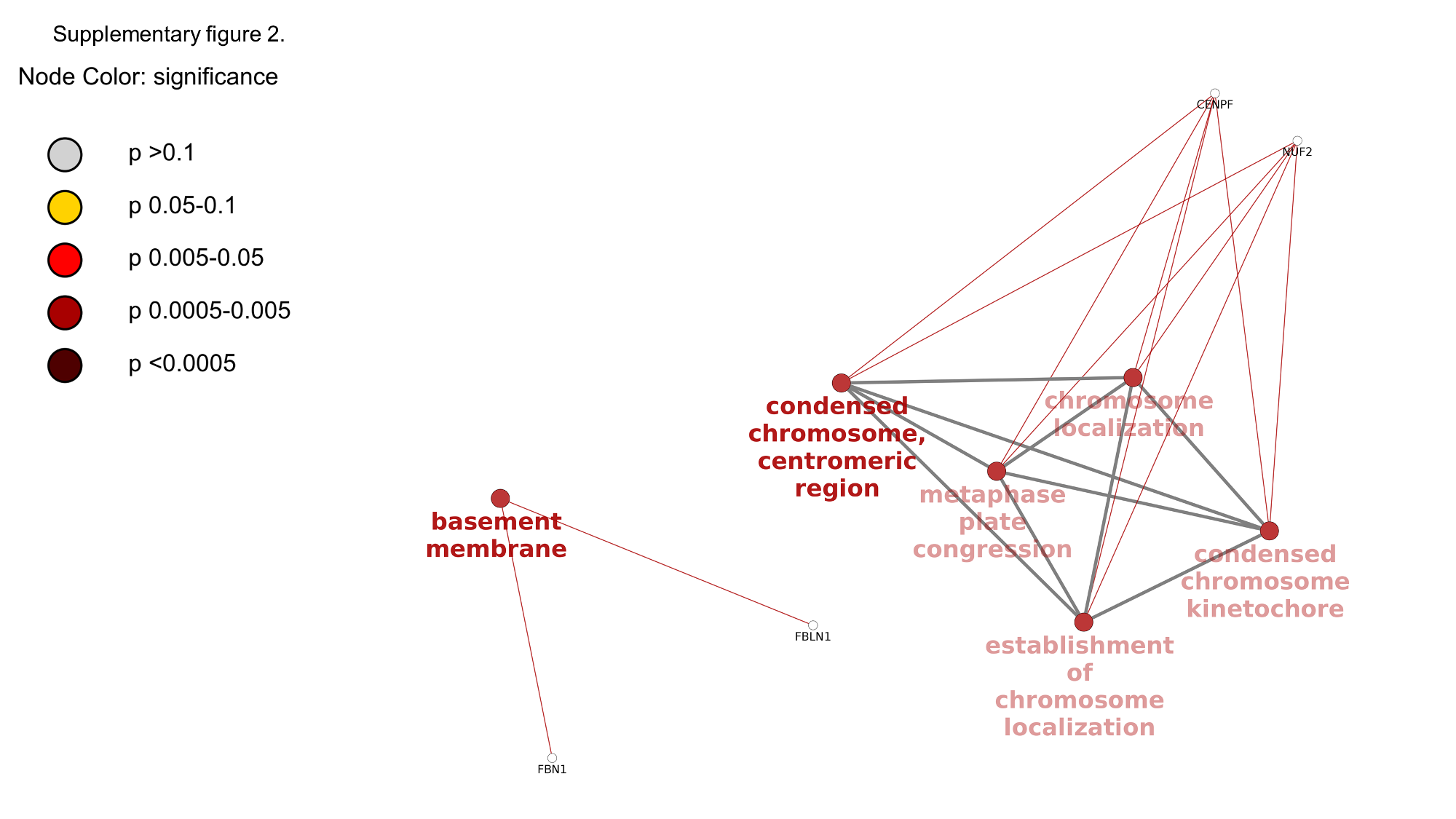

Supplement: S2 Fig — Differential gene expression analysis in LN across treatment groups showed DE genes in treatment group 6 versus 2. Gene ontology enrichment analysis of these genes (p<0.05) was conducted and results were visualized as a map of predicted connections among significantly enriched GO terms and related genes. (TIF) [file pone.0246695.s002.TIF]
